# Robot-Assisted Common Bile Duct Exploration in Trinidad & Tobago

**DOI:** 10.7759/cureus.30965

**Published:** 2022-11-01

**Authors:** Shamir O Cawich, Richard Spence, Ramdas Senasi, Vijay Naraynsingh

**Affiliations:** 1 Surgery, Port of Spain General Hospital, St. Augustine, TTO; 2 Surgery, Port of Spain General Hospital, St Augustine, TTO; 3 Radiology, Sunderland NHS Foundation Trust, Sunderland, GBR; 4 Clinical Surgical Sciences, Port of Spain General Hospital, St. Augustine, TTO; 5 Surgery, Medical Associates Hospital, St. Joseph, TTO

**Keywords:** common bile duct, laparoscopy, exploration, choledocholithiasis, gallstones

## Abstract

Although laparoscopic common bile duct exploration is a feasible and safe option for the operative management of choledocholithiasis, there has been a general reluctance to perform this procedure in Caribbean practice. This is largely because duct exploration is perceived to be difficult with laparoscopic instruments, and endoscopic retrograde cholangiopancreatography (ERCP) has become increasingly available. We report a case in which stones were extracted laparoscopically from the common bile duct, aided by the FreeHand® (Freehand 2010 Ltd., Guildford, Surrey, UK) robot, to show that the procedure is feasible and safe in the Caribbean environment.

## Introduction

Laparoscopic cholecystectomy is globally accepted as the gold standard operation to remove the gallbladder [1]. However, there is still debate on the ideal approach when common bile duct (CBD) stones are present.

In the era of open surgery, CBD exploration was routinely performed when stones were identified [2], but there was a general reluctance for duct exploration with laparoscopy because it was perceived to be difficult with laparoscopic instruments. This, combined with the increasing availability of endoscopic retrograde cholangiopancreatography (ERCP), relegated laparoscopic CBD exploration to only a few, experienced centers.

At the Port of Spain General Hospital, Trinidad & Tobago, a well-established hepatobiliary service exists [3-4]. In this setting, we routinely explore the CBD laparoscopically. We found that the FreeHand® (Freehand 2010 Ltd., Guildford, Surrey, UK) robot added value to this procedure and we report a case of laparoscopic common duct exploration aided by the FreeHand robot.

## Case presentation

A 36-year-old woman presented to the surgical clinic complaining of colicky right upper quadrant pain associated with nausea and vomiting. She gave a history of two prior hospitalizations for acute calculous cholecystitis, managed conservatively.

Trans-abdominal sonography confirmed the presence of multiple small gallstones within the gallbladder. There were no signs of acute inflammation. The common bile duct was 6mm in diameter, with no sonographic evidence of choledocholithiasis. Blood results, inclusive of liver function tests, were normal. Pre-operative gastroscopy was normal. Therefore, she was offered laparoscopic cholecystectomy.

**Figure 1:**
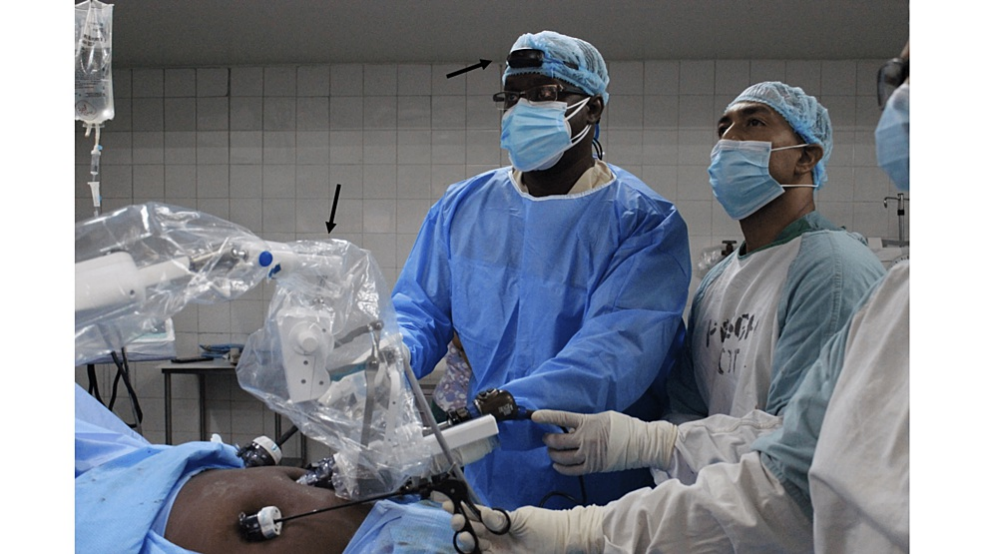
External view of the operating field The robotic arm (arrow) manipulates the laparoscope which is controlled by the surgeon with a head-mounted infrared communicator (arrow).

This patient was prepared for general anesthesia and taken to the operating room. Access to the peritoneum was secured with an open technique. A standard 10mm 30-degree laparoscope was passed across a 12mm port at the umbilicus and secured to the FreeHand® robotic arm. Three 5mm working ports were placed in the upper abdomen. The operating surgeon controlled the FreeHand robotic arm with a head-mounted infrared communicator, allowing full control of the intra-operative view (Figure 1).

**Figure 2:**
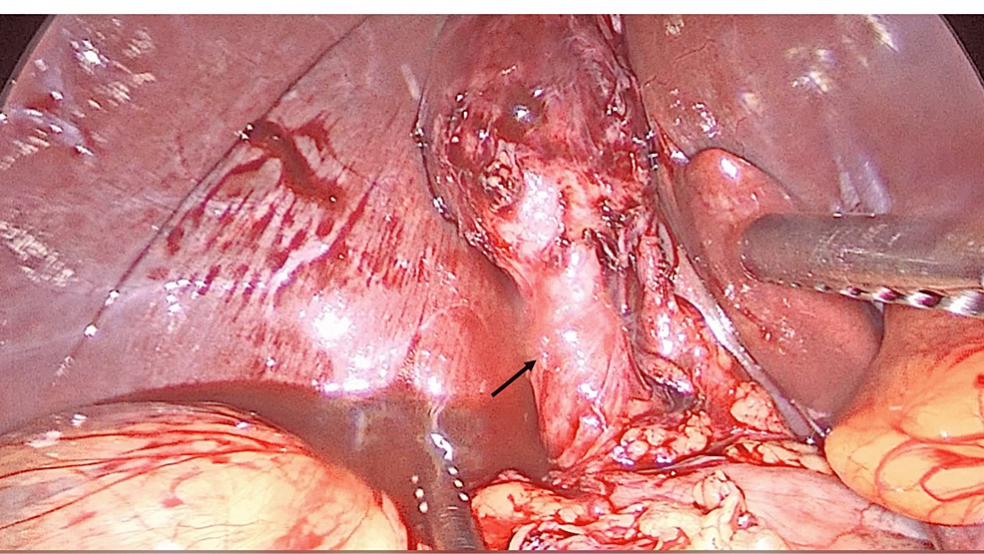
Internal view of the operative field This exposed and critical view allowed the surgeon to recognize that the cystic duct (arrow) was markedly dilated.

**Figure 3:**
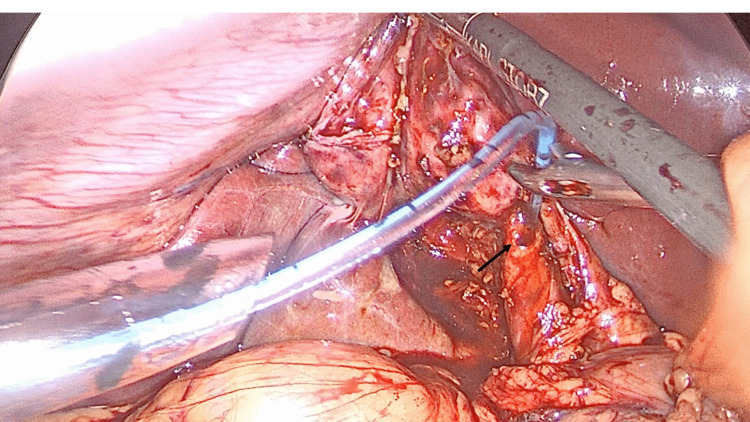
Operative cholangiography A needle holder is used to manipulate the infant feeding tube into the partially transected cystic duct (arrow) using a freehand technique.

**Figure 4:**
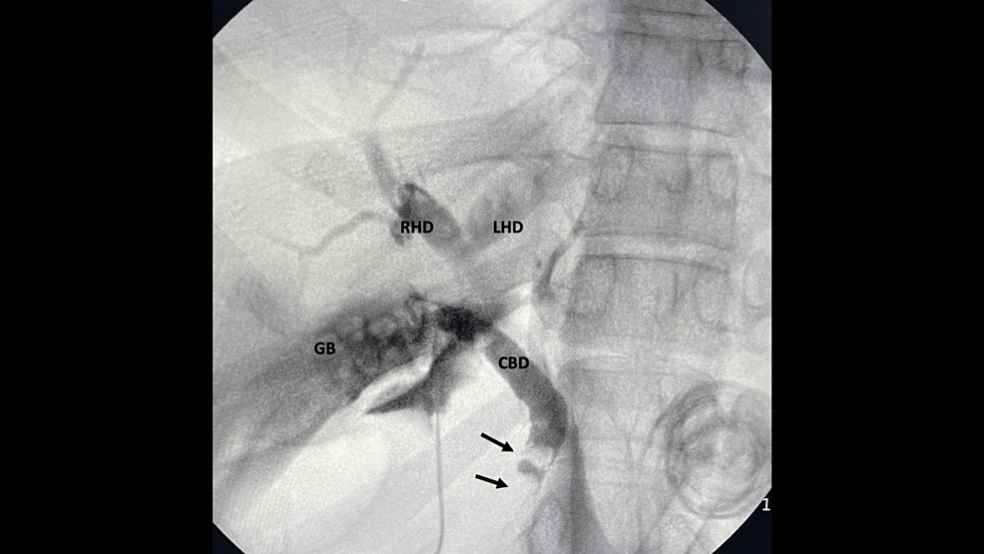
Fluoroscopic images taken during intra-operative cholangiography There are two filling defects (arrows) in the distal common bile duct (CBD), representing common duct stones. Multiple filling defects are also present in the gallbladder (GB), but the proximal CBD, left (LHD) and right hepatic ducts (RHD) are clear.

The operation proceeded by controlled dissection to demonstrate Strasberg’s critical view of safety. However, the cystic duct appeared dilated intra-operatively (Figure 2). Therefore, the decision was taken to perform an intra-operative cholangiogram. At this facility, a cholangiogram catheter passer is not available. Therefore, we performed cholangiography using an unguided technique (Figure 3). Here, the cystic duct was partially transected with scissors and a 5Fr infant feeding tube was manipulated using laparoscopic instruments without a guide into the opened cystic duct. The tube was advanced into the cystic duct and fixated in place with clips. This was used to instill 20mls of radio-opaque contrast medium, while fluoroscopic images were attained. Two unanticipated stones were identified in the distal common bile duct (Figure 4). There were no stones in the proximal extra-hepatic biliary tree.

**Figure 5:**
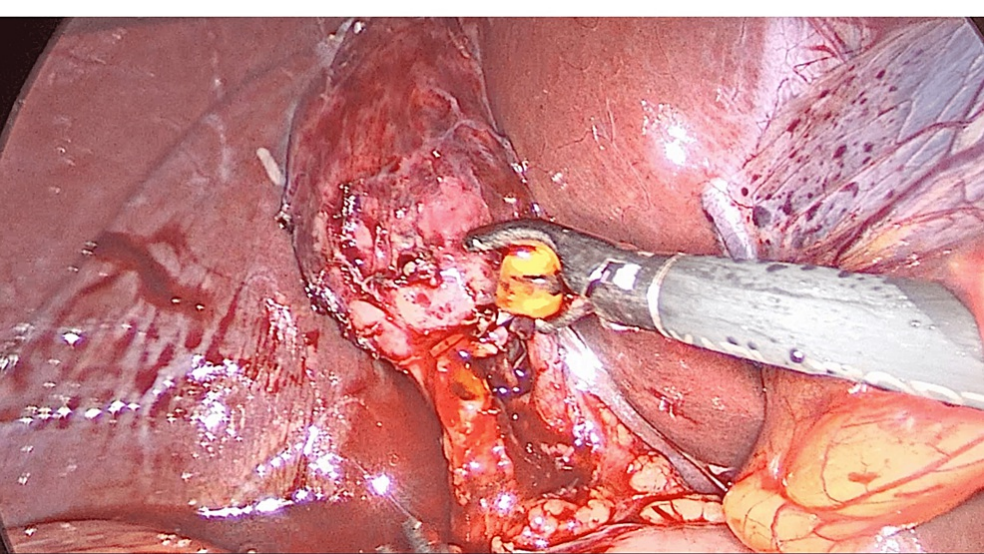
Gallstone extraction A gallstone being extracted once delivered into the cystic duct opening using a Fogarty catheter.

**Figure 6:**
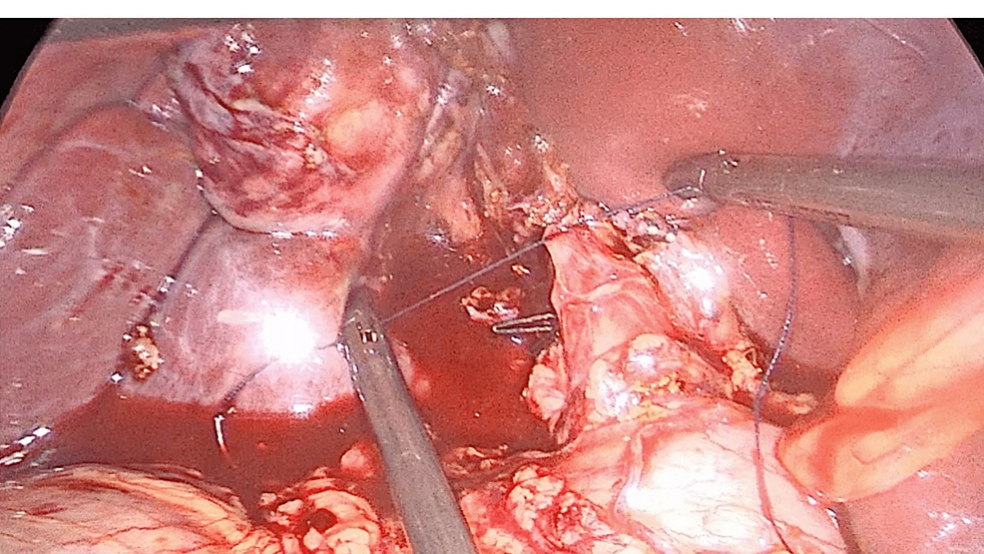
Cystic duct closure The dilated cystic duct stump was too large to be closed with clips and required suture ligation for secure closure.

After the detection of the common duct stone, a decision was taken to proceed with laparoscopic CBD exploration. We introduced a Fogarty catheter and used this to dilate the cystic duct. The CBD was then trawled with the Fogarty to return two large stones and significant amounts of sludge (Figure 5). After cholangiography to confirm complete stone removal, the dilated cystic duct stump was closed using 3-0 PDS sutures (Figure 6). The gallbladder was then dissected from the hepatic bed using cautery. There were no complications encountered and this patient was discharged from the hospital within 24 hours.

## Discussion

In patients undergoing laparoscopic cholecystectomy, common duct stones are present in 5% to 10% of cases [5]. With the increased availability of ERCP [6-7], laparoscopic exploration of the CBD has been largely relegated to sub-specialty services in high-volume centers. This has been reinforced by surgeons’ perceptions that laparoscopic CBD exploration is difficult to perform [8,9].

However, we must acknowledge that ERCP is associated with complications such as perforation, bleeding and pancreatitis [6-7]. These complications occur in 10% [7] to 11.1% [6] of persons undergoing ERCP in Caribbean practice. Moreover, patients would require two procedures, two exposures to anesthesia, and increased cumulative morbidity [10].

Alternatively, patients scheduled for laparoscopy may undergo intra-operative cholangiography, with a plan to proceed to duct exploration if stones are identified in the CBD. This would allow patients to be treated completely at a single sitting, limits the possibility of a negative ERCP, and avoids ERCP-related complications [10-12]. Pan et al. compared the outcomes of single-stage laparoscopic CBD exploration and cholecystectomy versus pre-operative ERCP and subsequent laparoscopic cholecystectomy in a meta-analysis with 1,757 patients with common duct stones across 13 studies [12]. They reported that there were significantly greater stone clearance rates (94% vs 90%) in the patients who underwent single-stage laparoscopic cholecystectomy with CBD exploration. In addition, there was a significantly lower cost of care, morbidity (7.6% vs 12%), retained stones (1.2% vs 7.9%), cumulative operating time (112 vs 132 minutes), and hospitalization (4.9 vs 6.6 days) in the single-stage laparoscopy group.

Despite the strength of the data in support of single-stage laparoscopic common duct exploration, most Caribbean surgeons still preferentially treat patients with choledocholithiasis by pre-operative ERCP followed by laparoscopic cholecystectomy [8]. This is largely due to a perception that laparoscopic CBD exploration is difficult. A survey of Caribbean surgeons revealed that laparoscopic CBD exploration was performed routinely by surgeons in only three out of 17 countries in the Anglophone Caribbean: Barbados, St Lucia, and Trinidad & Tobago. One of the main reasons touted was that their operating theatres were not prepared to facilitate this service as there were no cholangioscopes, catheter passers, or other specialized equipment.

At the Port of Spain General Hospital in Trinidad & Tobago, a well-established hepatobiliary service exists [3-4]. In this setting, we routinely explore the CBD laparoscopically. Despite the absence of specialized equipment, we have demonstrated that laparoscopic CBD exploration for common duct stones is feasible and safe. In our setting, we found that the FreeHand ® robot added value to this procedure. In our setting, an unguided technique was used to perform cholangiography and this had the potential to be interrupted by human camera persons. The passage of small catheters and instruments into small openings in the biliary tree also required precision. We found that the ability of the surgeon to be in control of his/her visual field afforded visual field stability that greatly aided in this task [13]. We advocate for the use of this technology, when available, for laparoscopic CBD exploration. Additionally, the head motions for robot control were intuitive as they were similar to those used to view a normal operating field [14].

There were disadvantages inherent to the use of the robotic arm: (1) additional operating time was required for docking of the robotic arm, (2) the docked robot limited instrument movement in the operating field, and (3) additional operating time was required to un-dock and re-dock the robot during fluoroscopy. However, we found that with attention to the robot docking position, there was acceptable instrument movement in the operating field. Also, time spent in docking/un-docking the robot was repaid by increased operator efficiency with the visual stability afforded by the robotic arm.

We also agree with other authors that it is important for the surgeon who wishes to undertake laparoscopic CBD exploration to have a working knowledge of biliary anatomy and variations [10,15], the ability to properly interpret intra-operative cholangiograms [16-17], an armamentarium of different approaches to duct exploration, stone extraction and closure [10,18,19], and the ability to suture laparoscopically [18,19].

## Conclusions

Single-stage, laparoscopic common bile duct exploration is feasible and safe in the Caribbean setting. The Freehand® system was of value since it eliminated human error to maintain steady uninterrupted vision, used intuitive motions that easily controlled the robot, and did not crowd the operating field. Before committing to laparoscopic common duct exploration, we advocate that surgeons have a working knowledge of biliary anatomy, be able to interpret operative cholangiograms, be familiar with different approaches to duct exploration, and can suture laparoscopically.
